# Evaluating AI chatbots for providing health advice on occupational sedentary behavior in the workplace

**DOI:** 10.3389/fpubh.2026.1965655

**Published:** 2026-09-30

**Authors:** Jingwei Zhang

**Affiliations:** Jiangsu Vocational College of Finance and Economics, Huai'an, Jiangsu, China

**Keywords:** artificial intelligence, Chatbot, digital health, occupational health, sedentary behavior

## Abstract

**Background:**

Office workers accumulate most of their waking h in sedentary behavior, yet scalable personalized occupational-health advice remains limited, and the quality of large language model (LLM)-based tools in this domain has not been rigorously examined.

**Objective:**

This study evaluated whether a domain-specialized chatbot system for providing health advice on sedentary behavior, integrating retrieval augmentation, a curated corpus, and safety guardrails, performs better than a general-purpose LLM and static educational materials.

**Methods:**

A sequential explanatory mixed-methods design was used. In the blinded expert evaluation, nine raters independently rated 60 responses across five quality dimensions. In the user survey, 654 eligible Chinese office workers interacted with the specialized chatbot and completed six perception scales (two adapted from established technology-acceptance frameworks and four developed for this study) plus open-ended feedback.

**Results:**

Inter-rater reliability was good for the composite score; in a case-level safety screen, no response from the specialized chatbot or the standard materials was flagged as potentially unsafe, whereas two Doubao responses were flagged by two or more raters. The specialized chatbot achieved the highest composite rating, followed by Doubao and standard materials. In a linear mixed-effects model and crossed random intercepts for rater and question, all Holm-adjusted pairwise contrasts were significant. Advantages of the specialized chatbot over Doubao were most pronounced on safety in symptom-management and special-population items. Among users, perceived ease of use was highest, whereas perceived safety and intention to use were rated comparatively lower. Frequent prior AI-chatbot use was associated with higher ratings on all six scales; after covariate adjustment the association persisted for five of the six scales but was attenuated and no longer significant for perceived safety.

**Conclusion:**

The specialized chatbot system received expert-rated accuracy and safety scores that did not differ significantly from standard educational materials, but exceeded standard materials on usefulness, comprehensibility, and completeness, and outperformed the general-purpose LLM on accuracy, safety, completeness, and the overall composite score. These findings concern response quality and immediate user perceptions, not behavioral or clinical outcomes. Nonetheless, users remained cautious about safety and intention to use after this brief exposure.

## 1 Introduction

Sedentary behavior is defined as any waking activity in a sitting, reclining, or lying posture with energy expenditure no greater than 1.5 metabolic equivalents. It is increasingly recognized as an occupational risk factor in the workplace (1). Previous studies have shown that sedentary behavior leads to a significant increase in all-cause mortality. Moreover, prolonged sitting is highly likely to induce cardiovascular diseases, type 2 diabetes, and musculoskeletal discomfort. Recent evidence from Chinese working populations suggests that urban white-collar employees spent an average of seven to nine hours of daily occupational sitting. Fewer than one-third of white-collar workers take breaks at the recommended frequency (2, 3). Sedentary behavior has become a priority for intervention in employee health promotion work.

Traditional workplace interventions include group lectures, printed brochures, and decision-point prompts. These measures moderately alleviate sedentary behavior but show poor long-term maintenance effects. Moreover, these initiatives cannot be widely promoted among populations. Mobile health (mHealth) applications and web-based programs improve reach but still deliver largely static content. They are unable to answer the specific situational questions raised by staff. Staff not only want to know the recommended break frequency, but also ask how to negotiate “stretch break time” with their supervisors. They are concerned about whether lower back pain after prolonged sitting requires medical attention. Moreover, they also ask about special conditions such as pregnancy, hypertension, or diabetes (4–6). Generative artificial intelligence (AI) has been expected to answer how to adjust activity recommendations for these situations.

Large language models (LLMs) have absorbed a large amount of biomedical and behavioral knowledge. Faced with open-ended questions, they can generate fluent natural-language responses. However, early evaluations of general-purpose chatbots for medical questions have produced mixed results. Their responses often contain occasional hallucinations, out-of-date medication instructions. In some scenarios involving “red flag” symptoms that require vigilance, they even provide highly inappropriate reassuring responses (7–9). The situation is different in areas such as postoperative education, urology, and chronic disease education. Researchers combined large language models with professional medical guideline corpora and adopted retrieval-augmented generation (RAG) technology. The results show that such “professional version” chatbots outperform unconstrained general large language models in both accuracy and safety (10–12). However, limited studies have targeted the specific domain of occupational sedentary behavior.

Beyond content quality, adoption of AI-based health advice depends on user trust, perceived usefulness, and perceived safety, as documented in empirical studies and systematic reviews of healthcare conversational agents (13–18). Technology acceptance research in healthcare shows that these perceptions are shaped by prior experience with AI, health literacy, and the perceived severity of the target condition (19, 20). In workplace settings, additional constraints apply: health advice must respect occupational hierarchies, avoid over-medicalizing, and align with national and employer health-promotion policies. If organizational norms do not support such adjustments, a clinically correct response that tells a worker to simply leave the desk for twenty min every h is not practical. Perceptions of usefulness therefore depend on both accuracy and relevance in specific contexts. Meanwhile, concerns about over-reliance, privacy, and algorithmic accountability in occupational health are also legitimate.

Against this background, this study evaluated a domain-specialized chatbot system—integrating retrieval augmentation, a curated knowledge corpus, and safety guardrails—that provides occupational sedentary-behavior advice to Chinese office workers. Two complementary perspectives were triangulated: a blinded, multi-professional expert rating of response quality against a general-purpose LLM (Doubao) and static standard materials, and an online user-experience survey of 654 office workers after a brief interaction with the specialized chatbot. This study focused on four research questions: how the specialized chatbot performs overall on expert-rated quality dimensions (RQ1); whether the three information sources differ across quality dimensions (RQ2); how performance varies across four clinically relevant question categories (RQ3); and how office workers perceive the chatbot on usability, usefulness, safety, trust, intention to use, and satisfaction, and whether these perceptions vary by prior AI experience (RQ4).

## 2 Materials and methods

### 2.1 Reporting guideline and ethical oversight

The study design, analysis, and reporting followed the Chatbot Assessment Reporting Tool (CHART) reporting guideline for chatbot health advice studies (9, 21, 22), with the checklist completed before data collection. This study followed the Declaration of Helsinki throughout the research. The protocol of this study was reviewed and approved by the Institutional Review Board of Jiangsu Vocational College of Finance and Economics (approval number JSU-2025-043). All the participants have provided informed consent. This study has not recruited participants younger than 18 years. Any identifying information of participants was not stored alongside survey responses. All expert rating data were processed by de-identification before statistical analysis. All survey data were collected through the Wenjuan Xing platform and stored on access-controlled, encrypted local drives accessible only to the author, with no personally identifying information linked to the stored responses. Chatbot conversations on the Coze platform were not retained beyond the study period. Expert ratings were collected through Qualtrics and exported as de-identified numeric files for analysis.

### 2.2 Study design overview

This study adopted a sequential explanatory design that triangulated blinded expert assessments with real-world user feedback. The expert phase, conducted under controlled conditions, served to anchor the quality assessment (RQ1–3) and mitigate user self-selection bias. The subsequent online survey captured end users' perceptions and open-ended feedback after autonomous interactions (RQ4). This design allows us to determine whether chatbots can theoretically generate high-quality responses. At the same time, it also reveals whether this quality can translate into users‘ perception of “usefulness” in practical application scenarios.

For RQ1, this study assessed overall performance based on descriptive statistics of composite scores. For RQ2, linear mixed-effects models with source as a fixed effect and crossed random effects for rater and question were used to identify source differences. For RQ3, descriptive profile analysis was employed, because clinical categories were predefined for validity coverage rather than experimental manipulation. For RQ4, psychometric validation (internal consistency) and subgroup analyses were used. Finally, the study quantified inter-rater reliability via ICC.

### 2.3 Information sources and fair comparison

Three information sources were compared. Both chatbot conditions were designed for on-demand question answering and health education rather than for self-monitoring or sustained coaching: neither system logged individual behavior, set personalized goals, or initiated proactive contact, so the comparison addresses the quality of advice content rather than behavior-change support. Group A was the specialized occupational-health chatbot developed by Coze (a general AI development platform open to the public, which is provided by ByteDance Company). It combines a fine-tuned dialogue model based on an open-source Chinese LLM with a retrieval-augmented generation (RAG) layer that retrieves from a curated corpus and constrains answers to retrieved evidence; a complete technical description of the system is provided in the following paragraph. Safety guardrails prevent medication dosing beyond package-insert language, require referral language for red-flag symptoms (chest pain, neurological deficits, unexplained weight loss, suicidality), and refuse out-of-scope queries. The retrieval corpus contained 68 documents totaling approximately 1.2 million Chinese characters, including WHO guidelines on physical activity and SB, China CDC workplace health promotion materials, the Chinese guideline for sedentary behavior in adults (2023), American College of Sports Medicine position stands, and selected narrative reviews on sedentary behavior and chronic disease (6, 23, 24). Temperature was set to 0.3 to favor deterministic outputs.

Technical description of Group A (reproducibility). The base dialogue model was GLM-4-9B-Chat, an open-source Chinese instruction-tuned large language model, hosted on the Coze platform; the model was adapted to the domain through supervised fine-tuning with approximately 1,200 curated question-answer pairs on occupational sedentary behavior, drafted from the guideline corpus by the author and reviewed by two occupational physicians who did not participate in the expert rating. No model weights were modified thereafter, and retrieval augmentation and safety guardrails were implemented at the platform orchestration layer rather than in the model weights. The knowledge corpus (68 documents, approximately 1.2 million Chinese characters) was assembled under explicit inclusion criteria: publication or endorsement by the WHO, the China CDC, Chinese national professional societies, or the American College of Sports Medicine; use of the most recent available edition; and exclusion of commercial and user-generated content. Where sources conflicted, the most recent national guideline took precedence, and superseded documents were removed from retrieval; the corpus was frozen for the entire study period (a quarterly review is planned for routine operation). Documents were segmented into passages of approximately 500 Chinese characters with a 50-character overlap, embedded with the platform's default text-embedding service, and stored in a vector database; for each user query, hybrid dense-and-keyword retrieval returned the top five passages, ranked by relevance score, which were inserted into the prompt context. The system prompt instructed the model to answer only from the retrieved passages, to state explicitly when the evidence was insufficient to answer, to use plain Chinese, to keep responses within 400 characters, and to append referral language whenever red-flag symptoms appeared. The safety guardrails (medication dosing restricted to package-insert language, mandatory referral wording for red-flag symptoms such as chest pain, neurological deficits, unexplained weight loss, or suicidality, and refusal of out-of-scope queries) were manually defined in consultation with one occupational physician and algorithmically enforced as pre- and post-generation filters. In the evaluated configuration, the chatbot did not display inline citations to retrieved sources to end users. The verbatim system prompt, retrieval configuration, and safety-rule definitions are provided in Supplementary Material S1. All evaluated responses from Groups A and B were generated in a single batch in June 2025. The 20 evaluation questions were finalized and archived before the system configuration was frozen, and were not used for corpus selection, prompt tuning, or any other development decision; the FAQ item pool was derived from independent elicitation interviews with office workers and guideline mapping (Section 2.4). Because one sampled generation per question and source was evaluated, all conclusions refer to the sampled responses rather than to the systems' average behavior across repeated generations; response-level stochasticity was constrained by the low temperature setting (0.3), but repeated-generation stability was not formally assessed and is acknowledged as a limitation. To keep decoding comparable across the two chatbot conditions, both systems used identical generation parameters: temperature 0.3, top_p 0.7, frequency penalty 0, presence penalty 0, and a maximum output length of 1,024 tokens, with a single response generated per question and source. The verbatim system prompt for Group A and the role prompt for Group B are provided in Supplementary Material S2.

Group B was a general-purpose LLM (Doubao 1.6, build version 2025-06-11, Volcano Engine). The two chatbot conditions deliberately used different base models: Group A required an open-weight model that could be hosted and orchestrated on the Coze platform with retrieval augmentation and platform-level safety filters, whereas Group B was intended to represent the strongest general-purpose Chinese assistant as actually deployed to consumers. Comparing two representative systems of each class was judged more informative for practice than an architecture-only comparison on a single base model; the resulting confound (base model and domain specialization are not isolated) is acknowledged in the Limitations (Section 4.6). To ensure a fair comparison rather than artificially handicapping the comparator, the model was invoked through the API with an explicit role prompt instructing it to act as an occupational health advisor specializing in SB and to provide evidence-based, cautious, plain-language answers, in Chinese, with referral language when symptoms suggested serious illness. Temperature was also set to 0.3; no function calling, browsing, or plugin access was enabled, and the model was frozen at the build version used throughout data collection to prevent silent updates from altering responses. Group C comprised static standard health education materials assembled from WHO fact sheets on physical activity and SB (24), China CDC workplace health promotion materials, and excerpts from the Chinese guideline for sedentary behavior in adults, reformatted as question-answer pairs by two occupational physicians who did not participate in expert rating. With respect to content overlap, the Group C source materials were drawn from the same guideline families as the Group A retrieval corpus (WHO, China CDC, and the Chinese guideline for sedentary behavior in adults), but the two document sets were not identical: Group A retrieved from the full 68-document corpus, which additionally included ACSM position stands and narrative reviews, whereas Group C was restricted to consumer-facing fact sheets and guideline excerpts. The comparison therefore reflects the real-world difference between a curated retrieval system and standard educational materials rather than a controlled content manipulation.

Each source responded to the same 20 frequently asked questions (FAQs) in Chinese. Responses were compiled into a single randomized list, with source labels replaced by alphanumeric codes; only the study coordinator held the key until all ratings were submitted. All responses were truncated at 400 Chinese characters to prevent length from confounding quality judgments.

### 2.4 FAQ test set construction

The FAQ test set comprised 20 questions. These questions evenly distributed across four predefined categories: risk perception (long-term health risks of occupational sitting, dose–response relationships); behavioral tips (break frequency, stretching, standing desks, walking breaks); symptom management (distinguishing ordinary soreness from red-flag symptoms, when to seek care for back pain, wrist pain, dizziness, headaches, and possible venous thromboembolism); and special populations (pregnant workers, workers with hypertension or diabetes, workers with chronic low back pain, older workers). The full English translation of the 20 questions appears in Supplementary Table S3. Item drafting followed a three-step procedure: (i) open-ended elicitation interviews with 12 office workers to generate candidate questions; (ii) topical binning by two occupational physicians to ensure coverage across the four categories; (iii) pilot testing with five additional clinicians to remove ambiguous or overly niche items. To avoid ceiling or floor effects, the item set was balanced between straightforward factual questions and more nuanced clinical scenarios in which safe referral language was required.

### 2.5 Expert evaluation

We specifically recruited nine experts to participate in the scoring. They come from three different professional backgrounds. This was done to achieve cross-validation. Detailed information about the nine experts is provided in Table 1. The expert panel consists of five women and four men. Their average professional experience was 9.8 years. Before the formal evaluation began, the team participated in a one-h online calibration meeting. The meeting clarified the specific operational criteria for the five scoring dimensions. The five-dimension instrument was developed specifically for this study: its dimensions were derived from the quality domains commonly used in previous evaluations of health-chatbot responses and mapped onto the reporting requirements of the CHART guideline, item wording was refined by two occupational physicians who also reviewed the scoring rubric for content validity, and the complete rubric, including the anchors for scores 1–6 and the operational distinction among medical accuracy, safety, usefulness, comprehensibility, and completeness, is provided in Supplementary Table S4. The experts also jointly discussed and trial-scored three test responses. These responses were all from outside the main test set. Subsequently, each rater conducted independent evaluations. They scored 60 responses through Qualtrics questionnaires. These 60 responses included three sources, corresponding to 20 common questions. To avoid order effects, the presentation order of responses was completely randomized. The expert evaluation was conducted fully online and asynchronously: each expert completed the 60-response questionnaire individually, at a time and place of their choosing and without a researcher present, following the same standardized written instructions (the scoring rubric and the operational dimension definitions described above); no time limit was imposed, and raters evaluated the same pre-generated, frozen response sets rather than interacting with live systems.

**Table 1:** Demographic and professional profile of the 9 expert raters.

Rater ID	Professional background	Qualification	Gender	Years of professional experience
R01	Occupational medicine	Board-certified occupational-medicine physician	Female	12
R02	Primary care	Primary-care physician with work-related consultation experience	Male	8
R03	Primary care	Primary-care physician with work-related consultation experience	Female	10
R04	Occupational medicine	Board-certified occupational-medicine physician	Male	14
R05	Workplace health promotion	Public health specialist (Master of Public Health)	Female	7
R06	Workplace health promotion	Public health specialist (Doctor of Public Health)	Male	6
R07	Occupational medicine	Board-certified occupational-medicine physician	Female	13
R08	Primary care	Primary-care physician with work-related consultation experience	Male	9
R09	Workplace health promotion	Public health specialist (Master of Public Health)	Female	9

The responses were rated across five dimensions (using a 1–6 Likert scale), with higher scores indicating better quality. The evaluation first focuses on medical accuracy and safety. We verified the accuracy of health risks, physiological mechanisms, and guideline recommendations. Simultaneously, we ensured the responses did not contain harmful advice. In high-risk scenarios, replies must include appropriate “warning” referral prompts and avoid providing false reassurance. Beyond clinical correctness, we also assessed other dimensions. For “usefulness,” this study emphasized whether the content provided practical advice for Chinese office workers. In terms of “comprehensibility,” this study required the language to be straightforward and easily understandable for non-specialist readers. As for “completeness,” responses cover all implicit aspects of the question. For example, they should offer immediate behavioral advice while also specifying when medical attention is necessary. In addition, this study had set up boxes next to each rating term so that the raters can provide specific feedback.

### 2.6 User survey

Participants in this study are office workers who primarily engaged in sedentary work with a weekly working time of no less than 30 h. The recruitment includes employees from different enterprises in three provinces (Hebei, Jiangsu, Yunnan) of China. Participants were invited by snowball sampling. Invitations were distributed via internal health communication channels, containing a brief overview of the research protocol and a QR code linking to the informed consent page. Because invitations were distributed through internal health communication channels and snowball referral, this non-probability strategy may have preferentially reached workers already interested in health or digital technologies; the implications for generalizability are addressed in the Limitations.

This study used Wenjuan Xing (a Chinese online survey platform) to host the questionnaire. Participants firstly completed demographic and occupational items, and two items on prior AI chatbot use (general health information use or workplace health chatbots). Participants were then invited to interact with the specialized chatbot (Group A) through a web interface for up to ten min. During this process, they could ask questions of their choice. After the interaction, they completed six perception scales. These scales included perceived ease of use, perceived usefulness, perceived safety, trust in the chatbot, intention to use, and overall satisfaction. In supplementary Table S5 presents the full item wording for all six perception scales. The perceived ease-of-use and perceived usefulness scales were adapted from the Technology Acceptance Model; the perceived safety, trust, intention-to-use, and satisfaction scales were developed for this study on the basis of the same construct definitions, drafted in Chinese, and translated into English using forward-back procedures by two bilingual researchers. All items were reviewed for content relevance and clarity by two occupational physicians who did not participate in the expert rating. No formal psychometric validation beyond internal-consistency analysis was undertaken, which is acknowledged as a limitation. All items used five-point Likert responses (1 = strongly disagree to 5 = strongly agree). Of the 734 questionnaires opened, 674 were submitted. Twenty submissions were excluded because they failed an embedded attention-check item, leaving 654 valid responses (97.0% of the 674 submitted questionnaires; 89.1% of the 734 opened). The median total completion time was 547.5 s (interquartile range 431–652 s; range 180–1,034 s; mean 544.2 s, SD 153.4 s; 5th-95th percentiles 289–786 s). Because the survey platform did not log individual chatbot sessions, the exact duration, number, and content of the questions posed by each participant could not be recorded; exposure was therefore heterogeneous and capped at ten min, and this is acknowledged as a limitation. As a partial remedy, total completion time, which subsumes the chatbot interaction because the interaction phase was embedded within a single continuous questionnaire flow, was used as an upper-bound proxy for exposure; its association with the perception scores is analyzed in Section 3.6 to assess whether exposure heterogeneity was related to the outcomes, and the qualitative free-text comments (*n* = 137) provide indirect indications of participants' interaction experiences. The user survey likewise proceeded without researcher presence: participants completed the questionnaire online, independently, and after reading standardized on-screen instructions; they interacted with the chatbot by asking questions of their own choice rather than responding to predefined scenarios, and, unlike the expert raters, they were not blinded to which tool they were using.

The 137 free-text comments were analyzed following an inductive, data-driven coding approach. The author read all comments, derived an open code list from a random subset of 30 comments, and then applied and iteratively refined the codes across the full set; the three final themes were established by grouping related codes and checking the resulting thematic structure back against the complete corpus. Because the coding was performed by a single researcher and the analysis was exploratory, the thematic findings should be regarded as indicative rather than independently corroborated; this is acknowledged as a limitation.

### 2.7 Statistical analysis

Analyses were conducted in Python (version 3.13) using pandas, SciPy, scikit-learn, and stats models. For expert data, an overall composite score per response was computed as the arithmetic mean of the five dimensions. Inter-rater reliability was assessed with the two-way random-effects, single-measurement, absolute-agreement intraclass correlation, ICC(2,1), at both composite and dimension levels, with 95% confidence intervals derived from the exact F-distribution method. ICC(2,1) was chosen because the nine raters can be regarded as representative of a broader population of occupational-health raters, each response was rated exactly once per rater, and absolute agreement rather than mere rank-order consistency is clinically meaningful, because raters' absolute safety judgments determine whether a response is considered safe. Per-source ICCs were additionally computed within each source's 20 items as sensitivity analyses. The statistical unit of the inferential analysis is the individual rating, nested within rater and question, and inference is made at the source level; because each of the nine experts rated the same 20 questions under all three source conditions, the 540 ratings constitute repeated measures clustered by both rater and question rather than independent observations.

Analyses for RQ1 (overall performance across sources): overall performance was first summarized with descriptive statistics of the composite score by source. The three sources were then compared with a linear mixed-effects model with source as a fixed effect and crossed random intercepts for rater and question, fitted for the composite score; adjusted pairwise contrasts between sources are reported as mean differences with 95% confidence intervals and Holm-corrected *p*-values. As a non-parametric sensitivity check, the Kruskal–Wallis test with Dunn's *post-hoc* pairwise comparisons (Bonferroni-corrected) was computed on the 540 composite ratings (Supplementary Table S6); because that procedure treats ratings as independent, it may overstate statistical significance and is therefore interpreted descriptively only.

Analyses for RQ2 (performance by quality dimension): the linear mixed-effects model described above was fitted separately for each of the five quality dimensions, and Holm-adjusted pairwise contrasts were used to identify which dimensions differentiated the three sources.

Analyses for RQ3 (performance across content categories): performance across the four predefined FAQ categories was analyzed descriptively, because the categories were designed for validity coverage rather than experimental manipulation. In addition, a pre-specified case-level safety screen was applied: a response was flagged as potentially unsafe when at least two of the nine raters assigned it a safety score of 3 or lower, a criterion that identifies responses about which independent experts expressed convergent concern; flags were tabulated by source and clinical category and cross-checked against raters' free-text comments.

Analyses for RQ4 (user perceptions): for the user survey, scale scores were computed as the mean of constituent items. Internal consistency was evaluated with Cronbach's alpha. Descriptive statistics were used to summarize perceptions. For pre-specified subgroup comparisons, we used Welch's *t*-tests and Cohen's d to contrast (i) participants who reported often or very often using AI chatbots for health information against those who did not, (ii) those with daily sitting ≥8 h against < 8 h, and (iii) those with self-reported chronic musculoskeletal conditions against those without. We also performed sensitivity analyses by excluding the 36 respondents (5.5%) whose completion times were below five min, and fitted covariate-adjusted linear regressions (adjusting for age, gender, education, and chronic-condition status) for the association between frequent prior AI-chatbot use and each perception scale, to assess robustness to inattentive responding and to confounding. To gauge whether heterogeneity in exposure was related to the outcomes, we additionally examined Spearman rank correlations between total completion time (an upper-bound proxy for interaction duration) and the six perception scales, and compared the fastest and slowest completion-time tertiles using Welch's *t*-tests and Cohen's d. In terms of confirmatory and exploratory status, the expert mixed-effects source comparisons, the dimension-level contrasts, and the case-level safety screen were the primary (confirmatory) analyses; the user-survey subgroup comparisons, the covariate-adjusted regressions, the sensitivity analyses, and the completion-time correlation analyses were secondary and exploratory, designed to probe robustness and to generate rather than to confirm hypotheses.

This study introduced a post-hoc power analysis using G^*^Power 3.1. For the user survey (*N* = 654, α = 0.05, and a two-tailed Welch's *t*-test), the statistical power exceeded 0.95 to detect small-to-medium subgroup differences (*d* = 0.25) on any perception scale. For the expert evaluation, no power claim is based on the 180 ratings per source, because these ratings derive from only nine raters and 20 questions and are not independent observations; the effective sample size is therefore substantially smaller than the nominal rating count, and precision for the expert comparisons is instead expressed through 95% confidence intervals from the mixed-effects model.

## 3 Results

### 3.1 Sample characteristics

Of the 654 valid user-survey respondents, 47.6% were men, 50.6% women, and 1.8% identified as non-binary or another gender; mean age was 34.5 (SD 7.9) years. The largest share held a bachelor's degree (60.1%), followed by associate degrees (18.2%) and postgraduate degrees (14.5%). Information technology and telecommunications (21.9%), manufacturing (16.5%), finance (16.1%), and government or public service (15.1%) were the most common industry sectors. Among the participants, mean weekly working time was 43.4 h, and mean daily occupational sitting was 7.6 h. These figures are consistent with national survey data, in which 53.0% of Chinese adults reported occupational sitting of more than four h per day (26). Self-reported chronic musculoskeletal complaints were present in 15.3%, hypertension in 12.1%, diabetes in 3.8%, and mental-health conditions in 3.4%. In terms of AI experience, 32.7% of respondents reported using AI chatbots often or very often for health-related information, whereas 13.6% reported never doing so; 12.7% reported regular prior use of workplace-specific health chatbots (see Table 2).

**Table 2:** Sociodemographic and occupational characteristics (*N* = 654).

Characteristic	Value
Age, years, mean (SD)	34.5 (7.9)
Weekly working hours, mean (SD)	43.4 (6.4)
Daily occupational sitting, hours, mean (SD)	7.6 (2.1)
Gender, *n* (%)	
Male	311 (47.6)
Female	331 (50.6)
Non-binary/other	12 (1.8)
Highest education, *n* (%)	
High school or below	47 (7.2)
Associate degree	119 (18.2)
Bachelor's degree	393 (60.1)
Postgraduate	95 (14.5)
Monthly personal income (CNY), *n* (%)	
< 4,000	59 (9.0)
4,000–8,000	197 (30.1)
8,000–15,000	249 (38.1)
15,000–25,000	111 (17.0)
>25,000	38 (5.8)
Industry sector, *n* (%)	
IT/telecommunications	143 (21.9)
Finance	105 (16.1)
Education	75 (11.5)
Healthcare	58 (8.9)
Manufacturing	108 (16.5)
Government/public service	99 (15.1)
Other	66 (10.1)
Self-reported chronic condition, *n* (%)	
None	389 (59.5)
Musculoskeletal	100 (15.3)
Hypertension	79 (12.1)
Diabetes	25 (3.8)
Mental-health condition	22 (3.4)
Other	39 (6.0)
AI chatbot use for health information, *n* (%)	
Never	89 (13.6)
Rarely	142 (21.7)
Sometimes	209 (32.0)
Often	138 (21.1)
Very often	76 (11.6)

### 3.2 Inter-rater reliability

Because all 60 responses were rated by the same nine experts, reliability was estimated with ICC(2,1) (two-way random effects, absolute agreement, single measurement). As summarized in Table 3, reliability was good for the composite score [ICC(2,1) = 0.93, 95% CI (0.89, 0.95)] and substantial to moderate across dimensions. Per-source composite ICCs, computed within the 20 items of each source as sensitivity analyses, were lower (see the note to Table 3). These within-source values reflect the restricted between-item variance once a single source is considered, which increases the relative contribution of rater idiosyncrasy. The high overall ICC therefore indicates good agreement across the full response set spanning all three sources, but it should not be read as uniformly strong agreement on every individual response within each source; consistent with the restricted within-source variance, raters were less consistent in discriminating among individual items within a source, an expected psychometric consequence of the narrower score range. For this reason, the primary inference in this study is made at the source level through the mixed-effects models, and item-level comparisons are interpreted descriptively.

**Table 3:** Inter-rater reliability of expert ratings [ICC(2,1), 9 raters × 60 responses].

Rated outcome	ICC(2,1)	95% CI
Composite quality score	0.93	[0.89, 0.95]
Comprehensibility	0.97	[0.96, 0.98]
Usefulness	0.91	[0.88, 0.94]
Safety	0.83	[0.77, 0.88]
Completeness	0.81	[0.75, 0.87]
Medical accuracy	0.77	[0.70, 0.84]

ICC(2,1) = two-way random-effects, single-measurement, absolute-agreement intraclass correlation. Per-source composite ICCs (sensitivity analyses, computed within the 20 items of each source): Group A 0.36 [0.21, 0.57]; Group B 0.37 [0.22, 0.59]; Group C 0.29 [0.15, 0.50]; dimension-level patterns were similar (e.g., medical-accuracy ICCs of 0.26, 0.54, and 0.21 for Groups A, B, and C, respectively).

### 3.3 Overall performance across information sources (RQ1)

The specialized chatbot (Group A) received the highest composite quality rating (*M* = 5.06, SD = 0.11), followed by Doubao (Group B, *M* = 4.47, SD = 0.13) and standard educational materials (Group C, *M* = 4.15, SD = 0.16). Because the ratings are repeated measures clustered by rater and question, the three sources were compared with a linear mixed-effects model with source as a fixed effect and crossed random intercepts for rater and question. All Holm-adjusted pairwise contrasts on the composite score were significant: Group A outperformed Group B [adjusted mean difference = 0.60, 95% CI (0.57, 0.62), *p* < 0.001), Group A outperformed Group C [0.91, 95% CI (0.89, 0.94), *p* < 0.001], and Group B outperformed Group C [0.31, 95% CI (0.29, 0.34), *p* < 0.001]. Figure 1 presents boxplots of the composite distribution across the three sources. The advantage of Group A over Group C was concentrated in usefulness, comprehensibility, and completeness, whereas the two sources did not differ significantly on medical accuracy or safety; these non-significant differences should not be interpreted as evidence of equivalence. In the complementary case-level safety screen, no response from the specialized chatbot (0/20) or the standard materials (0/20) was flagged as potentially unsafe, whereas two Doubao responses were flagged; the case-level analysis is presented below and in Table 4. A non-parametric sensitivity analysis (Kruskal–Wallis test with Dunn's *post-hoc* comparisons, Bonferroni-adjusted) on the 540 composite ratings confirmed the same ordering of sources [H(2) = 479.95, *p* < 0.001; all adjusted pairwise comparisons *p* < 0.001; Supplementary Table S6].

**Figure 1:**
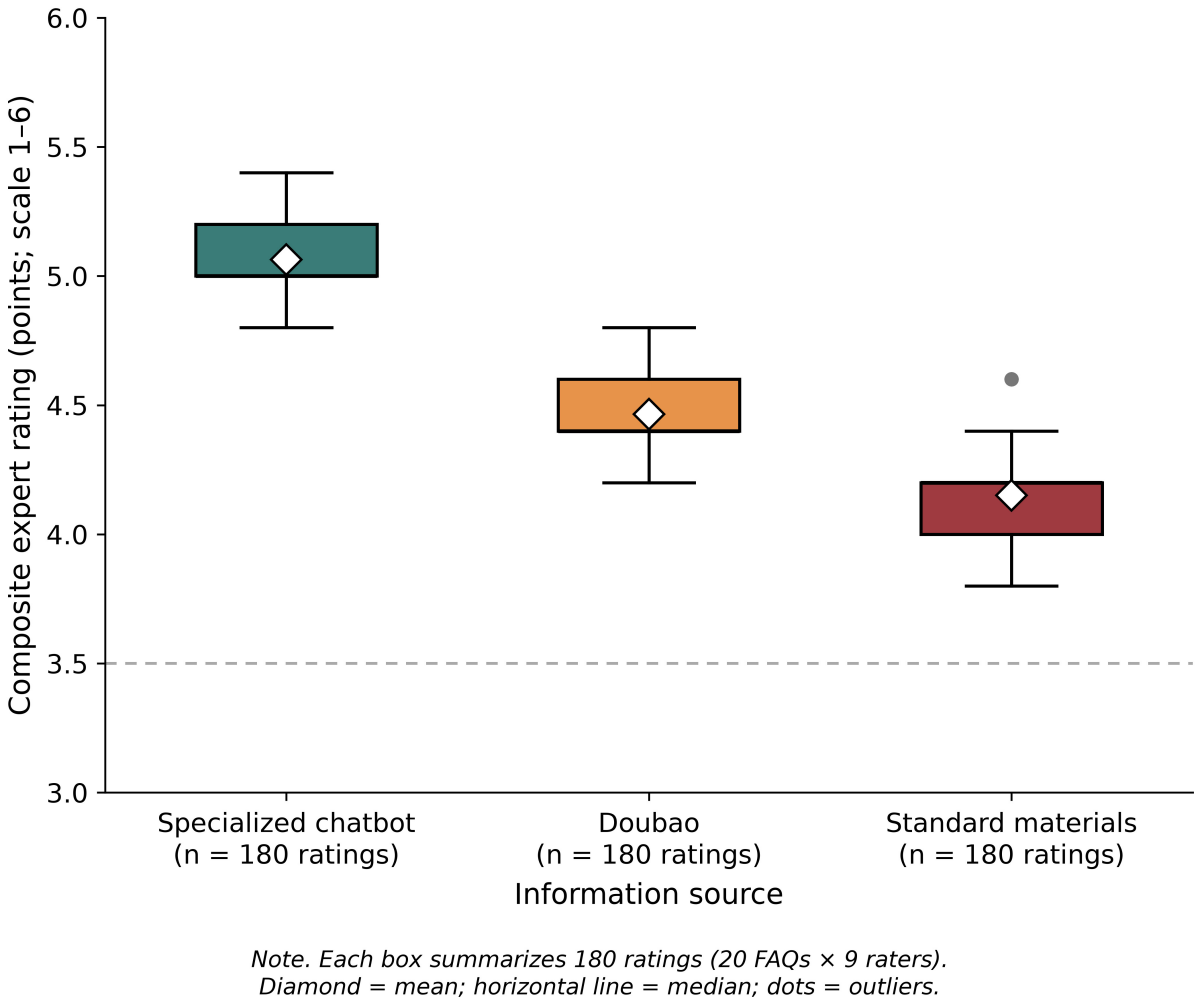
Distribution of composite expert ratings by information source. Boxplots show the distribution of the composite expert rating (points; scale range 1–6), computed for each of the 60 responses (20 FAQs × 3 information sources) as the mean of the five quality dimensions rated by nine experts (medical accuracy, safety, usefulness, comprehensibility, and completeness), yielding 180 ratings per source. Diamonds indicate means; horizontal lines within boxes indicate medians; box edges indicate the interquartile range; whiskers extend to observations within 1.5 × IQR of the box edges; dots indicate observations beyond this range. The dashed horizontal line at 3.5 marks the midpoint of the rating scale.

**Table 4:** Case-level safety flags by information source and clinical category.

Clinical category	Group A: specialized chatbot	Group B: doubao	Group C: standard materials
Risk perception	0/5	0/5	0/5
Behavioral tips	0/5	0/5	0/5
Symptom management	0/5	2/5	0/5
Special populations	0/5	0/5	0/5
Total (primary criterion: >=2 of 9 raters scored safety < =3)	0/20	2/20	0/20
Total (sensitivity criterion: any single rater scored safety < =3)	0/20	4/20	0/20

Case-level safety analysis. Beyond mean ratings, each of the 60 responses was screened against the pre-specified criterion of at least two of nine raters assigning a safety score of 3 or lower. No response from the specialized chatbot (0/20) or the standard materials (0/20) was flagged as potentially unsafe, whereas two Doubao responses (2/20) were flagged, both in the symptom-management category: one response to a question about low back pain after prolonged sitting, flagged by four of nine raters (panel mean safety rating 3.56), and one response about neck and shoulder stiffness after computer work, flagged by two of nine raters (panel mean 3.78). Raters' free-text comments attributed these flags to the absence of guidance on when to seek medical evaluation (missing referral language) and, for the back-pain response, to reassuring language about symptoms that can indicate more serious pathology. When the criterion was relaxed to any single rater, two further Doubao responses were flagged (one risk-perception item and one symptom-management item), and still no Group A or Group C response was flagged by any rater. These case-level results indicate that no evaluated response from the specialized chatbot was judged potentially unsafe, or as containing false reassurance or missing referral guidance, by more than one expert, whereas the safety deficit of the general-purpose LLM was concentrated in a small number of symptom-management responses rather than being uniform across items (Table 3). Because the expert evaluation was primarily quantitative, these flags are rating-based and should be read as a structured screening result rather than as a full clinical audit of response texts.

### 3.4 Performance by quality dimension (RQ2)

Looking at each dimension separately gives a clearer picture of how the three sources compare. For medical accuracy, all three scored above the midpoint: Group C (*M* = 5.13) was marginally ahead of Group A (*M* = 5.07), a small difference that did not reach conventional significance (adjusted mean difference = **–**0.06, 95% CI [**–**0.12, 0.00], *p* = 0.054), while Group B scored lowest (*M* = 4.17). On safety, Group A (*M* = 5.23) and Group C (*M* = 5.22) were statistically indistinguishable [adjusted mean difference = 0.01, 95% CI (**–**0.06, 0.08), *p* = 0.750], while Group B trailed at 3.96—largely because it sometimes skipped the referral language needed for symptom or special-population questions (Section 3.5). Group A clearly outperformed Group C on usefulness [5.03 vs. 3.41; adjusted mean difference = 1.62, 95% CI (1.56, 1.68), *p* < 0.001], comprehensibility [4.98 vs. 3.04; 1.94, 95% CI (1.91, 1.98), *p* < 0.001], and completeness [5.01 vs. 3.96; 1.04, 95% CI (0.99, 1.09), *p* < 0.001]. By contrast, Group A and Group B did not differ significantly on usefulness [0.03, 95% CI (**–**0.03, 0.09), *p* = 0.362], and Group B was marginally higher on comprehensibility [**–**0.03, 95% CI (**–**0.07, 0.00), *p* = 0.049], whereas Group A exceeded Group B on completeness [0.82, 95% CI (0.77, 0.87), *p* < 0.001]. The advantage over static materials likely comes from the chatbot's ability to generate responses that are specific to office contexts, action-oriented, and easy to follow. In the mixed-effects models, the fixed effect of source was significant for every dimension (Wald tests, all *p* < 0.001); see Table 5 for the full numbers.

**Table 5:** Mean (SD) expert ratings by information source and quality dimension.

Dimension	Group A: specialized chatbot	Group B: doubao	Group C: standard materials
Medical accuracy	5.07 (0.25)	4.17 (0.37)	5.13 (0.33)
Safety	5.23 (0.42)	3.96 (0.21)	5.22 (0.41)
Usefulness	5.03 (0.18)	5.01 (0.07)	3.41 (0.49)
Comprehensibility	4.98 (0.13)	5.02 (0.17)	3.04 (0.19)
Completeness	5.01 (0.07)	4.18 (0.39)	3.96 (0.19)
Composite (mean of five)	5.06 (0.11)	4.47 (0.13)	4.15 (0.16)

All ratings on a 1–6 Likert scale; higher = better. Linear mixed-effects models with crossed random intercepts for rater and question yielded a significant fixed effect of source on every dimension (Wald tests, all *p* < 0.001). Holm-adjusted pairwise contrasts were significant at *p* < 0.05 except for Group A vs. Group C on accuracy [**–**0.06, 95% CI (**–**0.12, 0.00), *p* = 0.054], Group A vs. Group C on safety [0.01, 95% CI (**–**0.06, 0.08), *p* = 0.750], Group A vs. Group B on usefulness [0.03, 95% CI (**–**0.03, 0.09), *p* = 0.362], and Group A vs. Group B on comprehensibility [**–**0.03, 95% CI (**–**0.07, 0.00), *p* = 0.049, a marginal difference favoring Group B].

### 3.5 Performance across content categories (RQ3)

Across content categories (see Table 6), Group A performed consistently at a high level, with mean composite scores of 5.05 for risk perception, 5.04 for behavioral tips, 5.06 for symptom management, and 5.10 for special-population items, combining guideline-correct dose information with contextual advice on negotiating breaks in Chinese office environments. Group B showed a relatively uniform profile across categories (*M* = 4.40–4.54) but received its lowest safety ratings in the symptom-management category, and rater comments pointed to multiple instances where Doubao produced responses that sounded clinically plausible yet failed to include appropriate referral language for questions involving persistent low back pain, neck and shoulder stiffness, and other symptoms that can indicate more serious pathology (see the case-level analysis in Section 3.3). Group C performed relatively better on risk-perception and symptom-management items (*M* = 4.20 and 4.22) than on behavioral tips and special-population items (*M* = 4.09 for both), reflecting the limitations of static materials in addressing idiosyncratic scenarios.

**Table 6:** Mean composite expert rating by source and FAQ content category.

Content category	Group A	Group B	Group C
Risk perception	5.05	4.54	4.20
Behavioral tips	5.04	4.46	4.09
Symptom management	5.06	4.40	4.22
Special populations	5.10	4.46	4.09

Each cell reports the mean composite score (mean of five dimensions, averaged over 5 FAQs × 9 raters = 45 observations). Higher = better.

### 3.6 User perceptions and variation by prior AI experience (RQ4)

The descriptive statistics and internal consistency of the user perception scales are shown in Table 7. All scales exhibited Cronbach's α values ranging from 0.83 to 0.87, indicating good internal consistency. The mean scores of all scales exceeded the scale midpoint of 3.0, ranging from 3.50 (intention to use) to 3.86 (perceived ease of use), suggesting that users formed generally positive yet not unreserved impressions after brief interaction. Among them, perceived ease of use scored the highest (*M* = 3.86, SD = 0.73), followed by perceived usefulness (*M* = 3.71, SD = 0.78), while trust (*M* = 3.57, SD = 0.82) and intention to use (*M* = 3.50, SD = 0.86) were relatively lower.

**Table 7:** Descriptive statistics and internal consistency of user-perception scales (*N* = 654).

Scale	Items	Mean	SD	Range	Cronbach's α
Perceived ease of use	4	3.86	0.73	1.00–5.00	0.85
Perceived usefulness	5	3.71	0.78	1.00–5.00	0.87
Perceived safety	4	3.66	0.79	1.00–5.00	0.83
Trust	4	3.57	0.82	1.00–5.00	0.85
Intention to use	4	3.50	0.86	1.00–5.00	0.84
Satisfaction	4	3.66	0.81	1.00–5.00	0.85

All items rated on a 1 (strongly disagree) to 5 (strongly agree) Likert scale; scale scores are the mean of constituent items. Perceived safety items were reverse-coded so that higher scores indicate greater perceived safety.

The correlation matrix (Table 8) shows that all scales are significantly positively correlated (*p* < 0.001), with correlation coefficients ranging from 0.35 to 0.65. The strongest correlation is between usage intention and satisfaction (*r* = 0.65), consistent with findings in technology acceptance literature. The moderate-to-strong correlations indicate that the scales are interrelated; however, because these associations are unadjusted and correlations alone do not establish that the six constructs are empirically distinguishable, no claim of discriminant validity is intended.

**Table 8:** Pearson correlations among user-perception scales (*N* = 654).

Scale	PEOU	PU	PS	Trust	ITU	Sat
PEOU	—					
PU	0.54	—				
PS	0.43	0.42	—			
Trust	0.43	0.47	0.54	—		
ITU	0.58	0.60	0.49	0.52	—	
Sat	0.57	0.54	0.47	0.51	0.65	—

PEOU, perceived ease of use; PU, perceived usefulness; PS, perceived safety; ITU, intention to use; Sat, satisfaction. All non-diagonal correlations were statistically significant at *p* < 0.001.

As noted in Section 2.7, the subgroup and qualitative analyses reported in this section are secondary and exploratory. Participants who reported often or very often using AI chatbots for health information (32.7% of the sample) rated the tool higher across all six scales than participants who used AI chatbots less frequently (see Supplementary Table S7). Differences were moderate in magnitude: perceived ease of use (Cohen *d* = 0.42), perceived usefulness (*d* = 0.48), perceived safety (*d* = 0.45), trust (*d* = 0.47), intention to use (*d* = 0.46), and satisfaction (*d* = 0.50). These statistics indicate that employees with more frequent prior AI-chatbot use reported more favorable perceptions; however, because these comparisons are unadjusted and cross-sectional, they describe an association rather than a causal effect, and confounding by factors such as digital literacy, health literacy, education, or age cannot be excluded. In covariate-adjusted linear regressions (adjusting for age, gender, education, and chronic-condition status), frequent prior AI-chatbot use remained independently associated with higher scores on five of the six scales (adjusted mean differences of 0.16–0.26 scale points, all *p* < = 0.01), whereas the association with perceived safety was attenuated and no longer statistically significant [adjusted difference = 0.06, 95% CI (**–**0.07, 0.19), *p* = 0.36], suggesting that the unadjusted difference on this scale partly reflects confounding by respondent characteristics. If the association is genuine, those lacking such experience may require more comprehensive onboarding and should carefully evaluate transparency before accepting health advice from chatbots. By contrast, participants reporting daily occupational sitting of eight h or more, and those with self-reported chronic musculoskeletal conditions, showed small and mostly non-significant differences in perceptions relative to their counterparts (all |d| < = 0.27; the largest difference was a higher intention to use among participants reporting eight or more h of daily occupational sitting, *d* = 0.27, while all other contrasts had |d| < = 0.15), suggesting that the tool was perceived similarly across users with different SB exposure and symptom status after this brief first encounter. Finally, as a proxy for exposure heterogeneity, total completion time was negligibly associated with all six perception scales (Spearman rho = 0.02–0.06, all *p* >= 0.14), and the fastest and slowest completion-time tertiles did not differ significantly on any scale (all |d| < = 0.17, all *p* >= 0.11); the perception results therefore do not appear to be driven by the amount of time participants spent in the survey flow.

According to the qualitative free-text comments (*n* = 137), this study summarized three themes (see Supplementary Table S8). First, participants appreciated the speed and conversational format (for example, one IT employee believed it was easier to get the chatbot's answers than searching on the website). Second, several users expressed skepticism about the tool's ability to give individualized advice for their specific medical conditions. This is consistent with the quantitative finding that safety and trust scored below ease of use. Third, users required additional features which are related with workplace. These requirements include integration with calendar reminders for break prompts, manager-facing materials, and the ability to log sitting time automatically.

## 4 Discussion

### 4.1 Principal findings

This study evaluated a domain-specialized chatbot system (integrating retrieval augmentation, a curated corpus, and safety guardrails) for occupational sedentary-behavior advice alongside an LLM (Doubao) and standard educational materials, using blinded expert ratings and user perceptions. Three principal findings emerged. First (RQ1 and RQ3), the specialized chatbot achieved the highest overall composite quality score, and it performed better than Doubao on safety in the symptom-management and special-population categories. Second (RQ2), static educational materials remained competitive on factual accuracy and safety but fell well short on usefulness, comprehensibility, and completeness; this indicates that the real value of generative AI in workplace sedentary advice lies not in repeating facts from guidelines, but in providing context-aware and actionable suggestions in plain language. Third (RQ4), although office workers found the chatbot easy to use, they remained cautious about the safety of its recommendations and their intention to rely on the tool when the advice involved health issues caused by prolonged sitting (such as lower back pain or the risk of blood clots); this cautious attitude was associated with less prior experience in using AI, although the cross-sectional design does not permit a causal interpretation.

In terms of composite score, the specialized chatbot outperformed both Doubao and static materials with a large effect size. However, the advantage was uneven across dimensions. On medical accuracy, Group A and Group C did not differ significantly (*p* = 0.054), with Group C in fact marginally higher descriptively; this absence of a significant difference should not be read as evidence of equivalence, but it is consistent with the expectation that curated static guidelines and a specialized system constrained to those guidelines achieve similar factual correctness. On safety, Group A and Group C were statistically indistinguishable (*p* = 0.750). This is because static guideline excerpts rarely produce hallucinations or exaggerate instructions (10). When safety is defined as the absence of harmful advice, their conservatism inherently becomes an advantage. Nevertheless, static materials show clear deficiencies in symptom management regarding special populations, where workers need tailored guidance. Therefore, the specialized chatbot did not differ significantly from curated static materials on accuracy or safety, while remaining statistically comparable to the general-purpose LLM on usefulness and comprehensibility and exceeding it on completeness and the overall composite score. The likely mechanism is that retrieval augmentation constrains generation to guideline-concordant content while preserving the capacity to tailor wording to the office context, so the specialized system combines the factual conservatism of static guidelines with the contextual adaptiveness of generative AI.

### 4.2 Comparison with prior evidence on specialized health chatbots (RQ1 and RQ2)

The findings align with a growing body of evaluations comparing specialized health chatbots with general-purpose LLMs, as synthesized in recent systematic reviews of healthcare conversational agents (17, 18). In urologic patient education (8), postoperative discharge instructions, and diabetes self-management, domain-specialized systems have generally matched or exceeded general-purpose models on accuracy and safety while outperforming static patient education on readability and engagement. The present results extend this literature to occupational sedentary behaviors, a domain characterized by lower acute risk than postoperative care but high population prevalence and substantial downstream chronic-disease burden (2, 27). In terms of accuracy, the specialized chatbot did not differ significantly from static materials in the present sample—although a non-significant difference does not demonstrate equivalence—a finding that echoes evaluations of consumer-facing chronic disease information chatbots. In these assessments, retrieval augmentation based on high-quality corpora has been shown to reduce hallucination rates compared to unrestricted generation.

International comparisons of workplace digital health interventions are informative. Systematic reviews of workplace interventions to reduce sitting have typically reported short-term reductions of 30–60 min per workday from multi-component programs that combine environmental change, prompts, and managerial support (4, 25, 28–32). Chatbot-based interventions have shown promise for weight management and physical activity, but evidence specific to occupational sedentary behaviors remains limited. The present study does not test behavioral outcomes, so it cannot establish whether the chatbot reduces sitting. Instead, it sets the necessary prerequisites for conducting such trials, namely that the information provided must be accurate, safe, and practical compared to existing alternatives.

### 4.3 Performance across clinical content categories (RQ3)

Group B (Doubao) scored lower in symptom-management and special-population items. Experts noted that Doubao occasionally omits referral statements for red flag signs or provides reassuring responses when a more cautious tone is warranted (33–35). For questions on pregnant employees and chronic disease patients, its answers seem reasonable but do not align with guideline recommendations. This result is consistent with prior reports of LLM behavior in clinical vignette evaluations (36). Role prompts did not eliminate these tendencies totally. It suggests that medical chatbots intended for practical application may benefit from combining retrieval over selected corpora with explicit safety guardrails that enforce referral prompts and reject out-of-scope queries. Because the present design compares complete systems rather than isolating the contribution of retrieval augmentation itself, these inferences apply to the system as implemented rather than to RAG specifically. This is especially critical when users are inclined to take chatbot responses as medically authoritative. For quality assurance, a category-stratified review process—prioritizing expert screening of symptom-management and special-population responses, where the general-purpose LLM's safety deficit was concentrated—may therefore be more efficient than uniform review of all outputs.

### 4.4 User perceptions and the role of prior AI experience (RQ4)

User-perception findings are consistent with technology acceptance models in healthcare (13, 37, 38). During this study, ease of use and usefulness were highest, which is consistent with previous studies in vaccine consultation and chronic disease self-management. The lower scores for trust, intention to use, and perceived safety are consistent with prior evidence. Previous studies suggest that both clinicians and patients are skeptical of AI-generated health content (39). However, these findings do not represent a failure of the advice produced by the specialized system. They reflect appropriate caution among users when they are confronting a new technology with health implications. In addition, the higher ratings among frequent AI users are consistent with earlier findings that prior experience of AI is associated with greater acceptance (13), although the present cross-sectional association cannot exclude confounding by digital literacy, health literacy, education, age, or general technology affinity. If this association is confirmed, prior experience would be a natural focus for onboarding. Another design implication concerns transparency: because the evaluated system did not display inline citations to its retrieved sources, users could not verify the evidential basis of the advice (Section 2.3), and making the underlying guideline passages visible would be a low-cost way to support informed trust.

### 4.5 Practical implications for workplace health promotion

This study provides three practical implications for workplace health promotion. First, domain-specialized chatbots of the kind evaluated here can be used as auxiliary tools in workplace health programs. However, the corpus must be strictly filtered and equipped with clear safety guardrails. More importantly, such systems should serve as a supplement to medical consultations but not a substitute. The evaluated system was rated highly on usefulness, but users' sense of safety and trust was moderately low. This is evident when advice involves sedentary-related lower back pain, venous thrombosis risks or chronic disease management. Therefore, chatbots should be defined as “initial information gateways”. They should lead users to seek assistance from health experts rather than serving as diagnostic tools independently (27, 34, 39).

Second, chatbots should be integrated into a comprehensive intervention system. Evidence shows that employees not only want to understand the risks of prolonged sitting. They also hope to receive calendar reminders, environmental adjustment suggestions, and relevant materials for management. This also confirms that behavioral change cannot be achieved without the support of organizations and the environment (40). If a chatbot can answer “how often one should take a break” but fails to assist employees in implementing these suggestions in a supportive environment, the practical impact in preventing disease caused by sedentary will be limited. More broadly, AI chatbots are also being integrated into non-clinical healthcare applications such as medical-equipment maintenance under Industry 5.0 principles (41), suggesting that structured evaluation of accuracy, safety, and usability, of the kind applied here, is relevant across a widening range of workplace AI tools.

Third, clear guidance must be provided for users who are not familiar with AI. These users have limited awareness of the health risks associated with prolonged sitting. They are struggling to determine when to adopt AI suggestions and when a medical referral is necessary. The subgroup differences in this study indicate that we need to explain the chatbot's operation mechanisms and limitations, and clearly inform when to turn to human medical advice.

### 4.6 Limitations

Several limitations should be considered. First, since this study focuses on content quality and user perception, rather than verifying whether the tool can effectively reduce sedentary behavior or improve health indicators, neither the expert evaluation nor the user survey tested behavioral outcomes. A randomized controlled trial with objectively measured sitting time would be required to assess effectiveness. Second, although backgrounds of the experts were deliberately varied in China, cultural and regional norms about occupational health and AI may limit transferability to other countries. Third, all the data collected in this study were cross-sectional. Therefore, causal claims about AI experience shaping perceptions are tentative. Unexplored factors (such as health literacy or technology enthusiasm) may lead to the subgroup differences observed. Fourth, because the specialized chatbot differs from the general-purpose comparator in multiple respects (base model, domain specialization, retrieval augmentation, curated corpus, and safety guardrails), the design cannot isolate the independent contribution of retrieval-augmented generation; all conclusions therefore apply to the system as implemented rather than to RAG *per se*. Relatedly, the Group A retrieval corpus and the Group C source materials, although drawn from the same guideline families, were not identical document sets (Section 2.3), so the comparison between the specialized system and standard materials reflects real-world differences in corpus breadth and format rather than a controlled content manipulation. Fifth, the user survey measured initial perceptions after a single brief interaction of up to ten min, during which participants asked heterogeneous questions; it does not measure long-term use, sustained adoption, clinical usefulness, or effectiveness, and the perception scores should be interpreted accordingly. Sixth, participants were recruited through snowball sampling distributed via internal workplace health communication channels, a non-probability strategy that may have over-represented workers already interested in health or digital technologies; the findings may therefore not generalize to Chinese office workers more broadly. Seventh, the perceived safety, trust, intention-to-use, and satisfaction scales were developed for this study and their psychometric properties were assessed only through internal consistency, and the qualitative analysis was conducted inductively by a single researcher in an exploratory manner, so the thematic findings are indicative rather than independently corroborated. Eighth, the survey platform did not log individual interaction sessions, so per-participant interaction duration and question patterns could not be reported; total completion time was therefore used as an upper-bound proxy for exposure and was not associated with the perception scores (Section 3.6). Ninth, only one sampled generation per question and source was evaluated; because generative-AI outputs can vary across repeated generations, the evaluation assessed the sampled responses rather than the full distribution of possible model outputs, and repeated-generation stability was not formally assessed, although stochasticity was constrained by the low temperature setting (0.3). Tenth, the specialized chatbot was implemented on a hosted platform (Coze); although all configuration-level details are reported in Section 2.3 and Supplementary Material S1, some low-level implementation details (for example, the exact embedding model) are platform-internal and could not be independently verified.

## 5 Conclusion

For providing health advice on sedentary behavior in the workplace, the specialized chatbot system evaluated in this study did not differ significantly from curated static guidelines in expert-rated accuracy and safety (a non-significant difference that does not establish equivalence), but significantly outperformed static materials on usefulness, comprehensibility, and completeness, and outperformed the general-purpose LLM (Doubao) on accuracy, safety, completeness, and the overall composite score. These conclusions concern response quality under the tested conditions; they do not isolate the independent contribution of retrieval augmentation, nor do they address behavioral or clinical outcomes. Workplace users rated the tool's ease of use positively after a single brief interaction, but remained cautious regarding the safety of the advice and their intention to use the tool when it came to health issues such as lower back pain and the risk of blood clots associated with prolonged sitting; this caution was associated with lower prior AI experience, although a causal interpretation is not warranted by the cross-sectional design. Domain specialization, combined with curated corpus retrieval and clear safety safeguards, represents a promising design for the responsible deployment of generative AI chatbots in occupational health contexts, although this single-system comparison cannot establish it as a necessary prerequisite. Therefore, this paper recommends that such tools should be positioned as supplementary information portals for workplace health programs, rather than as standalone diagnostic tools.

## Data Availability

The original contributions presented in the study are included in the article/Supplementary material, further inquiries can be directed to the corresponding author.
