# Supplementary material for: Evaluating AI chatbots for providing health advice on occupational sedentary behavior in the workplace
Source: Front Public Health. 2026 Sep 30;14:1965655. doi: 10.3389/fpubh.2026.1965655 (PMC13661802; doi:10.3389/fpubh.2026.1965655)
Supplement: Supplementary file 1 [file Supplementary_file_1.docx]

**Supplementary Material S1. Technical documentation of the specialized chatbot (Group A)**

This document provides the configuration-level technical details of the specialized occupational-health chatbot evaluated as Group A in the main manuscript (Section 2.3). It is organized as follows: S1.1 system architecture; S1.2 base model and fine-tuning; S1.3 knowledge corpus; S1.4 document segmentation and embedding; S1.5 retrieval configuration; S1.6 system prompt; S1.7 safety guardrails; S1.8 generation and evaluation configuration; S1.9 comparator configuration (Group B).

**S1.1 System architecture overview**

The specialized chatbot was implemented on the Coze platform (a general AI application-development platform provided by ByteDance). The system consists of three layers: (i) a dialogue layer based on GLM-4-9B-Chat with supervised fine-tuning for the occupational sedentary-behavior domain; (ii) a retrieval-augmented generation (RAG) layer that retrieves passages from a curated guideline corpus and inserts them into the prompt context; and (iii) an orchestration layer in which manually defined safety guardrails are algorithmically enforced as pre- and post-generation filters. All components were hosted on the Coze platform; no model weights were modified after fine-tuning, and retrieval augmentation and safety filtering were implemented at the platform orchestration layer rather than in the model weights.

**S1.2 Base model and supervised fine-tuning**

The base dialogue model was GLM-4-9B-Chat, an open-source Chinese instruction-tuned large language model. The model was adapted to the domain through supervised fine-tuning (SFT) with approximately 1,200 curated question-answer pairs on occupational sedentary behavior. The question-answer pairs were drafted from the knowledge corpus (Section S1.3) by the author and reviewed by two occupational physicians who did not participate in the expert rating. Fine-tuning was performed with the platform's standard SFT workflow (LoRA-based parameter-efficient adaptation); no model weights were modified thereafter.

**S1.3 Knowledge corpus: composition and inclusion criteria**

The knowledge corpus comprised 68 documents totaling approximately 1.2 million Chinese characters. Documents were selected under the following explicit inclusion criteria:

| **Criterion** | **Specification** |
| --- | --- |
| Publisher | Publication or endorsement by the World Health Organization (WHO), the Chinese Center for Disease Control and Prevention (China CDC), Chinese national professional societies, or the American College of Sports Medicine (ACSM) |
| Currency | Most recent available edition of each document at the time of corpus assembly |
| Exclusion | Commercial content, user-generated content, and materials without an identifiable authoritative publisher were excluded |
| Language | Chinese-language documents, or official Chinese translations of international guidance |
| Conflict resolution | Where sources conflicted, the most recent national guideline took precedence; superseded documents were removed from the retrieval index |
| Update policy | The corpus was frozen for the entire study period; a quarterly review is planned for routine operation |

**S1.4 Document segmentation and embedding**

Documents were segmented into passages of approximately 500 Chinese characters with a 50-character overlap between adjacent passages. Passages were embedded with the platform's default text-embedding service and stored in a vector database. The exact embedding model is platform-internal and could not be independently verified; this is acknowledged in the limitations (Section 4.6 of the main manuscript).

**S1.5 Retrieval configuration**

| **Parameter** | **Value** |
| --- | --- |
| Segmentation size | ~500 Chinese characters per passage |
| Segmentation overlap | 50 characters |
| Retrieval strategy | Hybrid dense-and-keyword retrieval |
| Number of passages retrieved per query | Top 5 |
| Ranking | Ranked by platform relevance score after hybrid fusion |
| Passage use | Top-ranked passages inserted into the prompt context as the sole authoritative evidence base |

**S1.6 System prompt**

The system prompt (Chinese original, followed by an English translation) instructed the model to answer only from retrieved passages and to apply the response-format and referral requirements enforced by the guardrails (Section S1.7).

**S1.6.1 Chinese original (verbatim)**

你是一名职业健康顾问，专门解答久坐行为相关的健康问题。请严格遵守以下规则：（1）仅依据提供的参考资料回答问题，不得使用资料之外的知识；（2）如果参考资料不足以回答问题，请明确说明证据不足，并建议用户咨询医生或职业健康专业人员；（3）使用通俗易懂的中文回答，避免不必要的专业术语；（4）回答控制在400字以内；（5）如用户描述出现胸痛、神经系统症状（如肢体麻木无力）、不明原因体重下降、自杀意念等警示症状，必须在回答中加入建议及时就医的转诊提示；（6）不得提供超出药品说明书范围的用药剂量建议；（7）对于超出久坐行为与职业健康范围的问题，请礼貌拒绝并说明范围限制。

**S1.6.2 English translation**

You are an occupational health advisor specializing in questions related to sedentary behavior. Follow these rules strictly: (1) Answer only from the provided reference passages; do not use knowledge outside the passages. (2) If the passages are insufficient to answer, state explicitly that the evidence is insufficient and advise the user to consult a physician or occupational health professional. (3) Use plain, easy-to-understand Chinese; avoid unnecessary technical terms. (4) Keep responses within 400 characters. (5) If the user describes red-flag symptoms such as chest pain, neurological deficits (e.g., limb numbness or weakness), unexplained weight loss, or suicidal ideation, the response must include referral language advising timely medical care. (6) Do not provide medication dosing advice beyond package-insert language. (7) Politely refuse questions outside the scope of sedentary behavior and occupational health and explain the scope limitation.

**S1.7 Safety guardrails**

The safety guardrails were manually defined in consultation with one occupational physician and algorithmically enforced at the orchestration layer as pre-generation and post-generation filters. The rule set was as follows:

| **Rule ID** | **Rule** | **Trigger** | **Enforcement mechanism** |
| --- | --- | --- | --- |
| G1 | Medication dosing restricted to package-insert language | Response attempts to give a medication dose | Post-generation filter checks dosing statements against a medication term list; non-conforming generations are regenerated with the rule restated |
| G2 | Mandatory referral wording for red-flag symptoms | User query or generated response mentions chest pain, neurological deficits, unexplained weight loss, suicidality, or other predefined red-flag terms | Pre-generation detection of red-flag terms in the query activates a mandatory referral clause appended to the system prompt; post-generation filter verifies that referral wording is present, otherwise the response is regenerated |
| G3 | Refusal of out-of-scope queries | Query falls outside occupational sedentary behavior and occupational health | Pre-generation scope classification; out-of-scope queries receive a standardized polite refusal explaining the scope limitation |
| G4 | Answer-only-from-retrieved-evidence | Every query | System prompt constraint (S1.6) plus instruction that insufficient evidence must be declared explicitly |
| G5 | Response length cap | Every query | Post-generation filter truncates or regenerates responses exceeding 400 Chinese characters |

The safety rules were therefore manually defined (content) and algorithmically implemented (enforcement), as stated in Section 2.3 of the main manuscript. In the evaluated configuration, the chatbot did not display inline citations to retrieved sources to end users.

**S1.8 Generation and evaluation configuration**

| **Parameter** | **Value** |
| --- | --- |
| Temperature | 0.3 |
| Generations per question | One sampled generation per question and source |
| Batch generation date | June 2025 (single batch, all evaluated responses from Groups A and B) |
| Test-set isolation | The 20 evaluation questions were finalized and archived before the system configuration was frozen, and were not used for corpus selection, prompt tuning, or any other development decision; the FAQ item pool was derived from independent elicitation interviews with office workers and guideline mapping (Section 2.4) |
| Repeated-generation stability | Not formally assessed; acknowledged as a limitation (Section 4.6). Conclusions refer to the sampled responses |
| top_p | 0.7 |
| Frequency penalty | 0 |
| Presence penalty | 0 |
| Maximum output length | 1,024 tokens |

**S1.9 Comparator configuration (Group B)**

Group B was a general-purpose LLM (Doubao 1.6, build version 2025-06-11, Volcano Engine), invoked through the API with an explicit role prompt instructing it to act as an occupational health advisor specializing in sedentary behavior and to provide evidence-based, cautious, plain-language answers in Chinese, with referral language when symptoms suggested serious illness. Temperature was set to 0.3. No function calling, browsing, or plugin access was enabled, and the model was frozen at the build version used throughout data collection to prevent silent updates from altering responses. Group C (static materials) comprised WHO fact sheets on physical activity and sedentary behavior, China CDC workplace health promotion materials, and excerpts from the Chinese guideline for sedentary behavior in adults, reformatted as question-answer pairs by two occupational physicians who did not participate in the expert rating. The verbatim role prompt for Group B is provided in Supplementary Material S2.
