# Supplementary material for: Evaluating AI chatbots for providing health advice on occupational sedentary behavior in the workplace
Source: Front Public Health. 2026 Sep 30;14:1965655. doi: 10.3389/fpubh.2026.1965655 (PMC13661802; doi:10.3389/fpubh.2026.1965655)
Supplement: Supplementary file 3 [file Supplementary_file_3.docx]

Supplementary Table S3. The 20 FAQ questions evaluated by the expert panel (English translation from Chinese)

| FAQ_ID | Category | Question |
| --- | --- | --- |
| 1 | Risk Perception | What health risks are associated with prolonged sitting at work? |
| 2 | Risk Perception | How many hours of daily sitting is considered harmful? |
| 3 | Risk Perception | Does sitting increase my risk of cardiovascular disease? |
| 4 | Risk Perception | Is sitting for long periods linked to type 2 diabetes? |
| 5 | Risk Perception | How does sedentary work affect back and neck pain? |
| 6 | Behavioral Tips | How can I reduce sitting time during office hours? |
| 7 | Behavioral Tips | How often should I take standing or walking breaks at work? |
| 8 | Behavioral Tips | Are sit-stand desks effective for reducing sedentary time? |
| 9 | Behavioral Tips | What simple exercises can I do at my desk to break up sitting? |
| 10 | Behavioral Tips | How can I build a sustainable habit of moving more at work? |
| 11 | Symptom Management | My lower back hurts after sitting all day. What should I do? |
| 12 | Symptom Management | I feel stiff in my shoulders and neck after computer work—what helps? |
| 13 | Symptom Management | How can I reduce eye strain from long screen sessions? |
| 14 | Symptom Management | I get tension headaches after long desk-bound workdays—why? |
| 15 | Symptom Management | My legs feel heavy after a full day of sitting, is that normal? |
| 16 | Special Populations | Is prolonged sitting safe during pregnancy? |
| 17 | Special Populations | What adjustments are recommended for older workers with arthritis? |
| 18 | Special Populations | Should people with hypertension manage sitting time differently? |
| 19 | Special Populations | How should workers with chronic lower back pain arrange their workstations? |
| 20 | Special Populations | Are there special sedentary-behavior recommendations for new office employees? |
