# Supplementary material for: Evaluating AI chatbots for providing health advice on occupational sedentary behavior in the workplace
Source: Front Public Health. 2026 Sep 30;14:1965655. doi: 10.3389/fpubh.2026.1965655 (PMC13661802; doi:10.3389/fpubh.2026.1965655)
Supplement: Supplementary file 5 [file Supplementary_file_5.docx]

Supplementary Table S5. Multi-item perception scales used in the user survey

| **Scale / Items** | **Wording (English translation from Chinese)** | **Mean** | **SD** | **Cronbach's α** |
| --- | --- | --- | --- | --- |
| **Perceived Ease of Use** (4 items, adapted from TAM) |  | 3.86 | 0.73 | 0.85 |
| PEOU1 | Learning to use this chatbot was easy for me. | 3.92 | 0.78 |  |
| PEOU2 | I found the interaction with this chatbot clear and understandable. | 3.85 | 0.81 |  |
| PEOU3 | It was easy to get this chatbot to do what I wanted. | 3.80 | 0.79 |  |
| PEOU4 | Overall, I found this chatbot easy to use. | 3.88 | 0.76 |  |
| **Perceived Usefulness** (5 items, adapted from TAM) |  | 3.71 | 0.78 | 0.87 |
| PU1 | This chatbot helped me understand the health risks of prolonged sitting. | 3.74 | 0.82 |  |
| PU2 | The advice from this chatbot was practical for my daily work routine. | 3.68 | 0.85 |  |
| PU3 | This chatbot provided information that I could act upon immediately. | 3.65 | 0.83 |  |
| PU4 | Using this chatbot improved my awareness of when to take breaks. | 3.76 | 0.80 |  |
| PU5 | Overall, I found this chatbot useful for workplace health advice. | 3.72 | 0.81 |  |
| Perceived Safety (4 items, self-developed; reverse-coded before analysis) |  | 3.66 | 0.79 | 0.83 |
| PS1 | I am concerned that this chatbot might provide misleading health information. | 3.70 | 0.84 |  |
| PS2 | I worry that using this chatbot could make me over-rely on AI instead of seeking professional help. | 3.62 | 0.86 |  |
| PS3 | I have concerns about my privacy when using this chatbot for health questions. | 3.71 | 0.82 |  |
| PS4 | I fear this chatbot might give inappropriate reassurance for symptoms that require medical attention. | 3.60 | 0.85 |  |
| **Trust in Chatbot** (4 items, self-developed) |  | 3.57 | 0.82 | 0.85 |
| Trust1 | I trust the accuracy of the health information provided by this chatbot. | 3.62 | 0.84 |  |
| Trust2 | This chatbot is reliable for general workplace health guidance. | 3.55 | 0.86 |  |
| Trust3 | I feel confident that this chatbot's recommendations are based on sound evidence. | 3.58 | 0.83 |  |
| Trust4 | I would trust this chatbot's advice enough to share it with my colleagues. | 3.52 | 0.87 |  |
| **Intention to Use** (4 items, self-developed) |  | 3.50 | 0.86 | 0.84 |
| ITU1 | I intend to use this chatbot regularly for workplace health questions. | 3.48 | 0.89 |  |
| ITU2 | I would recommend this chatbot to my coworkers. | 3.55 | 0.88 |  |
| ITU3 | I would use this chatbot if it were integrated into my company's health portal. | 3.52 | 0.87 |  |
| ITU4 | I plan to apply the advice from this chatbot in my daily work life. | 3.45 | 0.90 |  |
| **Overall Satisfaction** (4 items, self-developed) |  | 3.66 | 0.81 | 0.85 |
| Sat1 | I am satisfied with the quality of responses from this chatbot. | 3.64 | 0.84 |  |
| Sat2 | The chatbot met my expectations for a workplace health tool. | 3.60 | 0.85 |  |
| Sat3 | I was pleased with the overall interaction experience. | 3.71 | 0.82 |  |
| Sat4 | This chatbot is a good addition to workplace health resources. | 3.68 | 0.83 |  |

**Notes:**

- All items measured on a 5‑point Likert scale (1 = strongly disagree to 5 = strongly agree).
- **Perceived Safety** items were originally worded as concerns (higher score = higher concern); for scale scoring, responses were reverse‑coded (1↔5, 2↔4) before computing the mean, so that higher scores indicate greater perceived safety.
- Item-level means are presented for reference; scale scores in the main text are the mean of constituent items after reverse‑coding where applicable.
- All items were translated from Chinese using standard forward‑backward procedures; the English wording above is a translation of the final Chinese instrument.
