# Supplementary material for: Evaluating AI chatbots for providing health advice on occupational sedentary behavior in the workplace
Source: Front Public Health. 2026 Sep 30;14:1965655. doi: 10.3389/fpubh.2026.1965655 (PMC13661802; doi:10.3389/fpubh.2026.1965655)
Supplement: Supplementary file 6 [file Supplementary_file_6.docx]

**Supplementary Table S6. Kruskal–Wallis test and Dunn’s post-hoc comparisons on composite expert ratings (non-parametric sensitivity analysis)**

The analysis was computed on the 540 composite ratings (20 questions × 3 sources × 9 raters; 180 ratings per source). Composite score = arithmetic mean of the five quality dimensions per rating. Because the procedure treats the ratings as independent observations (which they are not; they are clustered by rater and question), it may overstate statistical significance and is interpreted descriptively only, as a sensitivity check on the mixed-effects model reported in the main manuscript (Sections 2.7 and 3.3).

| **Source** | **n** | **Median composite score** | **Mean rank** | **Dunn comparison** | **z** | **p (Bonferroni-adjusted)** |
| --- | --- | --- | --- | --- | --- | --- |
| Specialized chatbot (Group A) | 180 | 5.00 | 450.43 | A vs. B | 11.77 | < 0.001 |
| General LLM (Group B) | 180 | 4.40 | 256.84 | A vs. C | 21.05 | < 0.001 |
| Standard materials (Group C) | 180 | 4.20 | 104.23 | B vs. C | 9.28 | < 0.001 |

Kruskal–Wallis H(2) = 479.95 (tie-corrected), p < 0.001. All three Bonferroni-adjusted Dunn pairwise comparisons were significant at p < 0.001, confirming the same ordering of sources as the mixed-effects model: Group A > Group B > Group C.

Note. The Kruskal–Wallis test was applied to composite scores at the rating level (N = 540). Dunn’s post-hoc comparisons used the normal-approximation z statistic with Bonferroni correction for three pairwise comparisons.
