# Supplementary material for: Evaluating AI chatbots for providing health advice on occupational sedentary behavior in the workplace
Source: Front Public Health. 2026 Sep 30;14:1965655. doi: 10.3389/fpubh.2026.1965655 (PMC13661802; doi:10.3389/fpubh.2026.1965655)
Supplement: Supplementary file 7 [file Supplementary_file_7.docx]

Supplementary Table S7. Subgroup differences by prior AI experience

| **Scale** | Frequent AI users vs. others (unadjusted Cohen’s d) | **Interpretation** |
| --- | --- | --- |
| Perceived ease of use | 0.42 | Small-to-moderate |
| Perceived usefulness | 0.48 | Moderate |
| Perceived safety | 0.45 | Moderate |
| Trust | 0.47 | Moderate |
| Intention to use | 0.46 | Moderate |
| Satisfaction | 0.50 | Moderate |

Note. All comparisons are unadjusted (Welch’s t-tests) and cross-sectional; they therefore describe associations between frequent prior AI-chatbot use and perception scores rather than causal effects. All differences favor frequent AI users. Frequent users = participants who reported using AI chatbots for health information often or very often (32.7% of the sample). All Cohen’s d values were statistically significant (p < 0.001). These associations indicate that frequent prior AI-chatbot use is associated with more favorable perceptions across all six measured scales; confounding by factors such as digital literacy, health literacy, education, or age cannot be excluded (see Section 3.6 of the main manuscript).
