# Supplementary material for: Evaluating AI chatbots for providing health advice on occupational sedentary behavior in the workplace
Source: Front Public Health. 2026 Sep 30;14:1965655. doi: 10.3389/fpubh.2026.1965655 (PMC13661802; doi:10.3389/fpubh.2026.1965655)
Supplement: Supplementary file 8 [file Supplementary_file_8.docx]

Supplementary Table S8. Summary of qualitative themes from open-ended feedback (n = 137)

| **Theme** | **Description** | **Illustrative Quote** |
| --- | --- | --- |
| **Appreciation for speed and conversational format** | Participants valued the chatbot's quick, natural-language responses over traditional information-seeking methods. | "The answers were clearer and faster than searching a health website." — IT employee, age 31 |
| **Skepticism about individualized advice** | Users questioned whether the chatbot could tailor advice to their specific medical or personal circumstances. | "It gives general suggestions, but I'm not sure it really understands my specific situation." — Finance worker, age 42 |
| **Requests for workplace-specific features** | Participants desired practical integrations that would embed the chatbot into daily work routines. | "It would be helpful if it could sync with my calendar to remind me to take breaks, or let me log my sitting time automatically." — Manufacturing employee, age 36 |

Note. Thematic analysis was inductive and exploratory: the comments were read by a single researcher (the author), an open code list was derived from a random subset of 30 comments and then iteratively refined across the full set, and the three themes were established by grouping related codes and checking the resulting thematic structure against the complete corpus (Section 2.6 of the main manuscript). Because the coding was performed by one researcher and the analysis was exploratory, the themes are indicative rather than independently corroborated. Quotes are translated from Chinese for illustrative purposes.
